# Trichothecene Genotype Profiling of Wheat *Fusarium graminearum* Species Complex in Paraguay

**DOI:** 10.3390/toxins14040257

**Published:** 2022-04-05

**Authors:** Andrea Alejandra Arrua Alvarenga, Julio César Masaru Iehisa Ouchi, Cinthia Carolina Cazal Martínez, Juliana Moura Mendes, Adans Agustín Colmán, Danilo Fernández Ríos, Pablo David Arrua, Claudia Adriana Barboza Guerreño, Man Mohan Kohli, María Laura Ramírez, Ana Acuña Ruíz, María Magdalena Sarmiento, María Cecilia Ortíz, Adriana Nuñez, Horacio D. Lopez-Nicora

**Affiliations:** 1 Centro Multidisciplinario de Investigaciones Tecnológicas, Departamento Central, Campus Universitario San Lorenzo, Universidad Nacional de Asunción, San Lorenzo 111421, Paraguay; ccazal@rec.una.py (C.C.C.M.); jmendes@rec.una.py (J.M.M.); pdaa88@gmail.com (P.D.A.); claudiabarboza30.10@gmail.com (C.A.B.G.); 2 Departamento Central, Facultad de Ciencias Exactas y Naturales, Campus Universitario San Lorenzo, Universidad Nacional de Asunción, San Lorenzo 111421, Paraguay; dfernandez@facen.una.py (D.F.R.); acunaana544@gmail.com (A.A.R.); sarmiento2795@gmail.com (M.M.S.); chechiortiz11@gmail.com (M.C.O.); adrinugonza@gmail.com (A.N.); 3 Departamento Central, Facultad de Ciencias Químicas, Campus Universitario San Lorenzo, Universidad Nacional de Asunción, San Lorenzo 111421, Paraguay; jcmiehisa@qui.una.py; 4 Departamento Central, Facultad de Ciencias Agrarias, Campus Universitario San Lorenzo, Universidad Nacional de Asunción, San Lorenzo 111421, Paraguay; adans.colman@agr.una.py; 5 Cámara Paraguaya de Exportadores y Comercializadores de Cereales y Oleaginosas, CAPECO, Asunción 001206, Paraguay; mmkohli@gmail.com; 6 Instituto de Investigaciones en Micología y Micotoxicología (IMICO), Consejo Nacional de Investigaciones Científicas y Técnicas-Universidad Nacional de Río Cuarto (CONICET-UNRC), Rio Cuarto 5800, Argentina; mramirez@exa.unrc.edu.ar; 7 Department of Plant Pathology, The Ohio State University, Columbus, OH 43210, USA

**Keywords:** cereal, food safety, *Triticum aestivum*, mycotoxins

## Abstract

Paraguay is a non-traditional wheat-producing country in one of the warmest regions in South America. Fusarium Head Blight (FHB) is a critical disease affecting this crop, caused by the *Fusarium graminearum* species complex (FGSC). A variety of these species produce trichothecenes, including deoxynivalenol (DON) and its acetylated forms (3-ADON and 15-ADON) or nivalenol (NIV). This study characterized the phylogenetic relationships, and chemotype diversity of 28 strains within FGSC collected from wheat fields across different country regions. Phylogenetic analysis based on the sequence of elongation factor-1α gene (*EF-1α*) from 28 strains revealed the presence of four species in the FGSC: *F. graminearum sensu stricto, F. asiaticum, F. meridionale* and *F. cortaderiae.* Ten strains selected for further analysis revealed that all *F. graminearum* strains were 15-ADON chemotype, while the two strains of *F. meridionale* and one strain of *F. asiaticum* were NIV chemotype. Thus, the 15-ADON chemotype of *F. graminearum sensu stricto* was predominant within the *Fusarium* strains isolated in the country. This work is the first report of phylogenetic relationships and chemotype diversity among *Fusarium* strains which will help understand the population diversity of this pathogen in Paraguay.

## 1. Introduction

Paraguay is one of the few countries that produce wheat in one of the world’s warmest regions and export this cereal regularly [1]. In 2019, Paraguay produced 1.1 million tons of wheat on 430,000 hectares and exported over 460,000 tons of grain to Brazil and other countries [1]. Unfortunately, wheat production in Paraguay is often threatened by several biotic and abiotic factors. Among the biotic limiting factors, FHB is one of Paraguay’s most important fungal diseases affecting wheat production [2]. FHB is caused by members of the *Fusarium graminearum* species complex (FGSC) [3], which can produce toxins for human and animal health and affect several sectors of the wheat supply chain [4]. The fungal species in the FGSC causing FHB produce trichothecenes that are strong phytotoxins and act as virulence factors to facilitate tissue colonization on sensitive host plants [5,6].

Currently, 16 monophyletic species have been identified within the FGSC [7] comprising, *F. austroamericanum* (lineage 1), *F. meridionale* (lineage 2), *F. boothii* (lineage 3), *F. mesoamericanum* (lineage 4), *F. acacia-mearnsii* (lineage 5), *F. asiaticum, F. graminearum sensu stricto* (lineage 7), *F. cortaderiae* (lineage 8), and eight additional monotypic lineages, including *F. brasilicum, F. vorosii, F. gerlachii, F. aethiopicum, F. ussurianum, F. nepalense, F. louisianense,* and US Gulf Coast population of *F. graminearum* [7,8,9,10,11,12,13].

Each *Fusarium* species has a specific profile of toxic secondary metabolites and a distinction can be made between chemotype I strains producing deoxynivalenol (DON) and (or) its acetylated derivatives and chemotype II strains producing nivalenol (NIV) and (or) 4-acetyl niva-lenol (4-ANIV). In addition, within the DON chemotypes, chemotype IA producing 3-ADON and chemotype IB producing 15-ADON were distinguished [14,15].

The species *F. graminearum sensu stricto* and *F. asiaticum* produce 3-ADON, 15-ADON, and NIV. All other species of the FGSC produce mainly NIV and (or) 3-ADON [16] as well as some trichothecene variant forms (15-acetyldeoxyvalenol, 3-acetyldeoxynivalenol, and nivalenol), which are species-specific and associated with pathogenicity [17]. Trichothecene gene cluster has been recognized as a 26-kb segment of DNA constituted of three loci in *F. graminearum* and *F. sporotrichioides*: a single-gene TRI101 locus, a two-gene TRI1-TRI16 locus, and a twelve-gene locus. These genes function in the biosynthesis, regulation, and transport of the mycotoxins across the plasma membrane [18,19]. TRI13 and TRI7 are the most important genes to determine the production of DON or NIV genotype [20].

There is a large diversity in the FHB causing species in the Southern Cone region of South America. For example, the Argentina *F. graminearum* populations from wheat are genotypically diverse and belong to *F. graminearum sensu stricto;* populations from Argentina were like those populations from Brazil with the same haplotype [21,22]. High genotypic diversity with significant differences within subpopulations and high gene flow would indicate that isolated strains from Argentina are part of the same unique and widespread population [23,24].

While *F. meridionale*, *F. asiaticum*, *F. graminearum*, *F. cortaderiae*, and *F. austroamericanum* have been reported to be associated with FHB in Brazil [22,25,26,27], *F. graminearum sensu stricto* was the most frequently isolated species in Uruguay [22,28].

Argentina, Brazil, and Uruguay represent the largest wheat-producing region in the Sothern Cone, where *F. graminearum* genotypes producing 15-ADON are predominant [27]. However, genotypes producing NIV, 3-ADON have also been reported [21,25,26,27,29,30,31,32,33,34]. To date, there is no genotypic information on *Fusarium* species isolates from Paraguay.

Since the 1970s, no epidemics of *Fusarium* wilt have been documented in Paraguay, and there are no data on the toxin production potential of the species present in the fields. In 1972 and 1975, two FHB and foliar blights epidemics caused wheat farmers to lose as much as 70% of their production, resulting in severe economic losses [35]. Previous studies have reported *F. graminearum* as the prevalent fungus producing DON mycotoxin in Paraguay [2,36,37,38,39].

Given the lack of data on genotypes and their association with the potential trichothecene production in Paraguay, this study aimed to determine the species composition and trichothecene genotypes of a set of strains collected from wheat fields. 

## 2. Results

### 2.1. Identification of Fusarium Strains to Species Level

**Figure 1:**
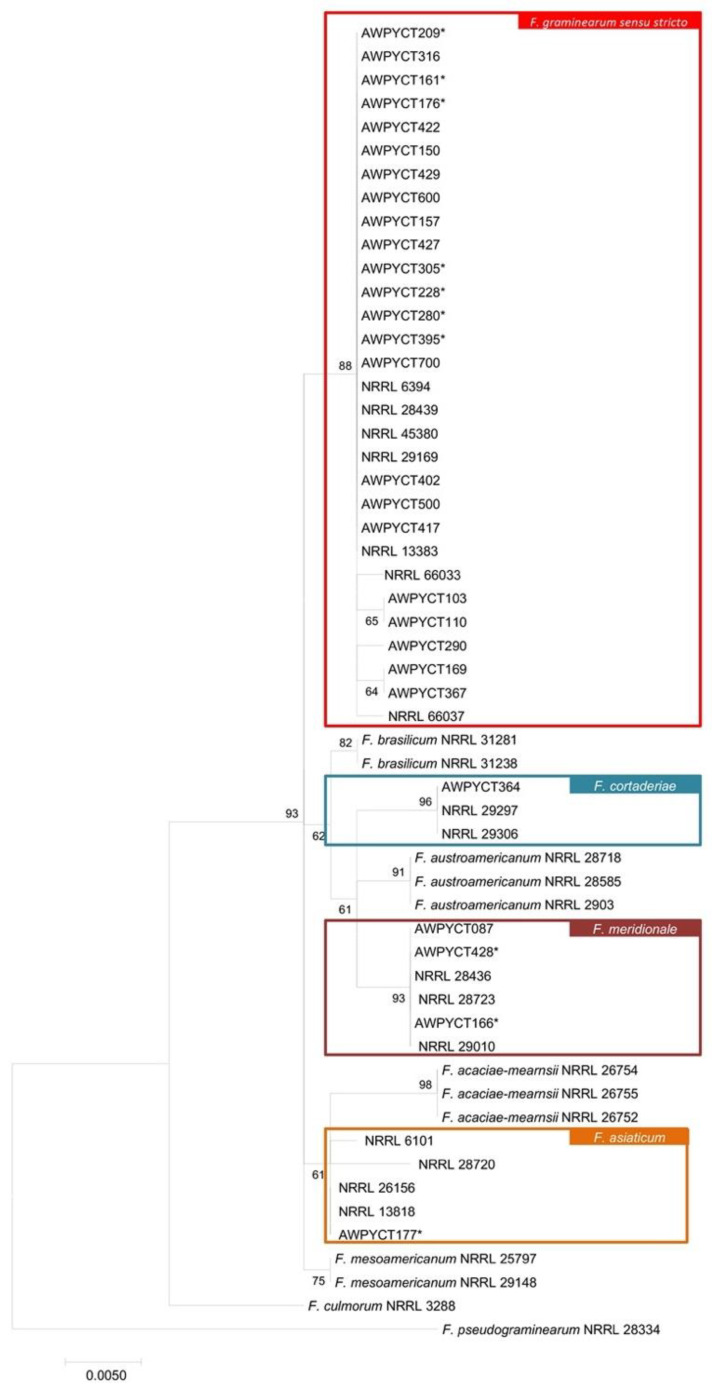
Maximum likelihood phylogeny of *Fusarium graminearum* Species Complex based on the alignment of *EF-1*α gene. Bootstrap values (percentage, based on 1000 replications) are shown on branches. The reference sequence *Fusarium* spp. was downloaded from the National Center for Biotechnology Information. *F. pseudograminearum* and *F. culmorum* were used as outgroups. “*” Strains from Paraguay.

Genetic polymorphisms in the DNA sequence of *EF-1α* were analyzed for 28 *Fusarium* strains isolated in this study to identify their species [8]. Based on the phylogenetic analysis of the *EF-1α* gene, 23 strains were grouped as *F. graminearum sensu stricto* and confirmed by the presence of the reference strain NRRL66037 [40]. In addition, a single strain AWPYCT087 belonged to *F. cortaderiae* together with the reference strain NRRL 29,297 [41]. The remaining three strains formed a monophyletic clade with *F. meridionale* reference strain NRRL 28,436 [3], and one strain AWPY177 belonged to *F. asiaticum* together with the reference strain NRRL6101 [3] (Figure 1).

### 2.2. Trichothecene Genotypes

Trichothecene genotypes of 10 selected strains were determined based on PCR amplification of *TRI3* and *TRI12* genes [42]. All *F. graminearum* strains, eight in our study, were 15-ADON chemotype. Otherwise, the two *F. meridionale* strains and the *F. asiaticum* strain were NIV chemotypes. These results indicate that all isolates studied have the potential capability to produce trichothecene mycotoxin.

## 3. Discussion

This work is the first phylogenetic and molecular analysis to determine the trichothecene genotype of FHB strains prevalent in FGSC populations collected from wheat fields in Paraguay.

The species belonging to FGSC are known to possess three specific profiles of trichothecene production, including nivalenol (NIV) and its acetyl derivatives, DON, and primarily 3-acetyldeoxynivalenol, 3-ADON and DON and primarily 15-ADON [43,44].

Based on the *EF-1*α gene sequences of wheat isolates, we were able to identify four species within FGSC. Of the examined strains, 82% belonged to *F. graminearum s.s.*, 11% to *F. meridionale*, 3% to *F. asiaticum* and 3% to *F. cortaderiae*. Concerning the trichothecene genotypes, all *F. graminearum s.s.* strains have a 15-ADON genotype, and the two strains of *F. meridionale* and one strain of *F. asiaticum* exhibit the NIV genotype.

Several studies have been carried out to identify FGSC genotypes associated with FHB in wheat, barley, rice, and maize in the Southern Cone Region, including Argentina, Brazil, Uruguay, and Paraguay (Table 1).

The results of the present study support findings from Argentina, where all 112 *F. graminearum* s.s isolates obtained from 28 locations belonged to the 15-ADON chemotype [45]. However, they coincide better with a three-year Brazilian study conducted on the barley fields in the State of Rio Grande do Sul. In this study, the authors reported the presence of three species within the FGSC. All 15-ADON strains were identified as *F. graminearum s.s.*, while all NIV strains were identified as *F. meridionale* [22,34], similar to our findings.

In another extensive study carried out in barley and wheat fields in the State of Paraná, Brazil, the authors determined that within the FGSC, *F.* *graminearum s.s.* with 15-ADON genotype was dominant (63%), followed by *F. meridionale* NIV genotype (23.1%), *F. cortaderiae* NIV (7%) or 3-ADON (2.6%) genotypes, and *F. austroamericanum* (3.8%) 3-ADON genotype [46].

Similarly, *F. graminearum s.s.* was the most frequently isolated species (97%) in Uruguay, although *F. cortaderiae* and *F. austroamericanum* were also identified [21]. The authors also reported 15-ADON (95%) as the predominant chemotype, followed by 3-ADON (3%) and NIV (2%). Most *F. graminearum s.s.* showed 15-ADON chemotype, and *F. austroamericanum* and *F. cortaderiae* isolates were 3-ADON and NIV chemotypes, respectively. Another four-year study from Uruguay confirmed that all the *F. graminearum* isolates presented the 15-ADON genotype. While *F. cortaderiae, F. asiaticum* and *F. brasilicum* isolates shared the NIV type, the single *F. austroamericanum* isolate had the 3-ADON type [28].

Therefore, the species composition of FGSC in the Southern Cone region (Argentina, Brazil, Uruguay, and Paraguay) is predominated by the presence of *F. graminearum* isolates with 15-ADON genotype. Other species, such as *F. meridionale, F. asiaticum* and *F. cortaderiae*, are of NIV genotype, and *F. austroamericanum* of 3-ADON genotype [43]. These results coincide with reports from North America, central Europe, southern Russia, and some parts of Asia where the 15-ADON genotype dominates but different from northern Europe, where 3-ADON dominates [5].

The continuous survey and surveillance of genotypes and chemotypes of the FHB pathogens are critical to determine the risk of pathogenic and toxigenic genotypes [47] and improve the FHB management strategies, especially under a climate change scenario [30]. To explain genotype distribution in different geographic regions, hypotheses based on grain/seed shipment, international trade, long-distance spore transportation, and environmental conditions have been proposed [5].

It must be emphasized that a variable FGSC population with different genotypes can adapt to different ecological environments, such as the hosts, temperature regime, crop rotation schedule, and agronomic management. Although 3-ADON-producing strains have been reported to be more aggressive than the 15-ADON population in susceptible wheat, the former isolates exhibited a greater capacity for DON production than the 15-ADON isolates. Given the toxicological differences between DON and NIV, it is crucial to monitor the population of genotypes and to determine the chemotypes of strains present in any geographical region [5]. Even so, in wheat, DON is an important virulence factor, and strains that produce DON are more virulent than those that do not. However, other specific aspects of the pathogen related to virulence must be considered [48,49]. In strains of different chemotypes and genotypes, comparable accumulation of both acetyl derivatives could be because acetyl derivatives biosynthesis is regulated by temperature [50].

The need to carry out long-term studies with a larger number of fungal strains from the wheat-producing region of Paraguay is well understood. In addition, the analysis of the distribution of FGSC and trichothecene genotypes in the cereal crops will help to understand the relationship between disease and mycotoxin contamination observed in the country. Such studies will also help develop effective management strategies to control the disease and mycotoxin contamination [51]. Our results place Paraguay at par with other countries in the region, mainly because of the fungal populations’ toxicogenic potential.

To our knowledge, this is the first study that used *Fusarium* strains from Paraguay to analyze the phylogenetic relationships and genotype diversity. Therefore, this study helps to understand the population diversity of FGSC in the country.

## 4. Conclusions

Phylogenetic analysis of 28 strains revealed the presence of four species within FGSC in wheat cultivated in Paraguay: *F. graminearum s.s.*, *F. asiaticum*, *F. meridionale* and *F. cortaderiae.*

Of the ten selected strains for trichothecene genotype analysis, all *F. graminearum s.s.* strains belonged to the 15-ADON genotype, the two *F. meridionale* strains and *F. asiaticum* strain presented the NIV genotype. As a result, the 15-ADON genotype of *F. graminearum sens s.s.* is predominantly found within the *Fusarium* strains isolated from wheat. 

This work is the first study to use *Fusarium* strains from Paraguay to analyze the phylogenetic relationships and chemotype diversity. We believe it essential to understand the population composition of FGSC in the country.

## 5. Materials and Methods

### 5.1. Fungal Isolates

Based on the morphological characters of the fungi, 28 isolates (strains) were identified as belonging to the FGSC and confirmed by sequencing *translation elongation factor-1 alpha* (*EF-1α*) [8]. The strains were collected from commercial wheat fields in four locations: Itapúa, Alto Paraná, Caazapá, and Canindeyú, representing major production regions of Paraguay. These were preserved as spore suspensions in 15% glycerol frozen at −80 °C [27] in the Collection of Cultures of Microorganisms of the National University of Asunción (CCM-UNA).

### 5.2. DNA Isolation

DNA was extracted with cetyltrimethylammonium bromide (CTAB) method from cultures grown in PDA for one week at 22 ± 1 °C. The resulting mycelia were harvested by plates’ surface scraping and stored frozen at −20 °C until ground and extracted with CTAB [32]. The DNA obtained was quantified using a DS-11-DeNovix spectrophotometer and was stored at −25 °C in a freezer.

### 5.3. Phylogenetic Analysis

For phylogenetic analysis, *EF-1 α* gene was amplified and sequenced using EF-1 and EF-2 primers (Table 2). Agarose gels of 0.8% concentration were made in 0.5× TBE (Tris borate EDTA) Buffer and run at 90 V, 100 mA for 60 min. Amplification products were compared to a 100 bp molecular weight marker, EZ Load™ 100bp Molecular Ruler (#170-8352) [52]). For gel staining, the 1× Diamond red intercalator, Diamond™ Nucleic Acid Dye from Promega, was used [53]. The gels obtained were visualized using a Gel Documentation System, UV light in Gel Doc EZ–Biorrad [54]. The amplified products were sequenced by the automatic pyro-sequencing method, Sanger dideoxy sequencing method, in Macrogen, Korea [55]. *EF-1 α* DNA sequences of 28 strains, together with known NRRL strains of FGSC from the GenBank, were aligned using ClustalW algorithm of MEGA 7.0.26 software [56]. The phylogenetic tree was constructed using the Maximum Likelihood method with 1000 bootstrap replicates. The best-fit model of molecular evolution was selected based on Bayesian Information Criterion scores [57].

### 5.4. Trichothecene Genotype Determination

The primers used for PCR-based identification of mycotoxin biosynthetic genes are presented in Table 2. The trichothecene genotypes of the strains were determined in a PCR reaction with primers for *TRI3* and *TRI12* alleles [42,58]. 

PCR reactions included 20 ng of genomic DNA as a template in a 50 μL reaction composed of 1× reaction buffer, 2 mM MgCl2, 1.25 U Taq DNA polymerase (Promega), 0.2 mM dNTPs, and 2 μM of each primer. PCR was performed in a thermal cycler (BioRad) with an initial denaturation step at 95 °C for 2 min; 30 cycles of 94 °C (1 min), 55 °C (1 min), and 72 °C (3 min); and a final extension step at 72 °C for 10 min [58]. PCR products were separated by electrophoresis on 2% agarose gels. Gels were stained, photographed, and further analyzed [32].

## Figures and Tables

**Table 1:** Presence and prevalence of *Fusarium* genotypes in the Southern Cone Region.

		Presence			Prevalence		
Country	Matrix	NIV	3-ADON	15-ADON	15-ADON	Year	Reference
Argentina	Wheat						
Argentina	Wheat	+	+	+	+	2011	[32]
Argentina	Wheat	+		+	+	2017	[29]
Argentina	Wheat			+	+	2014	[45]
Argentina	Durum wheat	+		+	+	2017	[30]
Uruguay	Wheat	+		+	+	2013	[31]
Uruguay	Wheat	+	+	+	+	2013	[21]
Uruguay	Wheat	+	+	+	+	2013	[28]
Uruguay	Wheat	+		+	+	2018	[33]
Brazil	Wheat	+		+	+	2012	[25]
Brazil	Barley	+	+	+	+	2011	[34]
Brazil	Wheat and Barley	+	+	+	+	2020	[46]
Paraguay	Wheat	+		+		This study	

(+) indicates the presence and prevalence of a particular trichothecene genotype.

**Table 2:** Primers used in this study.

Primer Name	Gene	Sequence 5′–3′	Reference
3CON	*TRI3*	TGGCAAAGACTGGTTCAC	[44]
3NA	*TRI3*	GTGCACAGAATATACGAGC	[44]
3D15A	*TRI3*	ACTGACCCAAGCTGCCATC	[44]
3D3A	*TRI3*	CGCATTGGCTAACACATG	[44]
12CON	*TRI12*	CATGAGCATGGTGATGTC	[44]
12NF	*TRI12*	TCTCCTCGTTGTATCTGG	[44]
12-15F	*TRI12*	TACAGCGGTCGCAACTTC	[44]
12-3F	*TRI12*	CTTTGGCAAGCCCGTGCA	[44]
EF-1	*EF-1α*	ATGGGTAAGGARGACAAGAC	[3,8]
EF-2	*EF-1α*	GGARGTACCAGTSATCATGTT	[3,8]
